# Advances in Surrogate Neutralization Tests for High-Throughput Screening and the Point-of-Care

**DOI:** 10.1021/acs.analchem.5c00666

**Published:** 2025-03-04

**Authors:** Simon Streif, Antje J. Baeumner

**Affiliations:** † Institute of Analytical Chemistry, Chemo- and Biosensors, University of Regensburg, Universitaetsstrasse 31, 93053 Regensburg, Germany

## Introduction

Serological testing has long played a crucial role in disease management and clinical diagnostics. Both infection with viruses and vaccination mediate a humoral immune response, including the generation of specific antibodies. The presence of antibodies can therefore be used qualitatively to detect recent or past infections or quantitatively to determine the immune status of a patient. Antibody profiles generated by different viruses vary and show differences for infection versus vaccination, as the latter often uses only one specific antigen rather than the whole virus. Furthermore, while some vaccinations result in long-term immunity, others require booster shots every few years, or even yearly. Hence, the necessity and benefits of serological testing are typically tailored to the respective viruses or diseases.

Diseases preventable through vaccination include, e.g., hepatitis A, influenza, SARS, chickenpox, measles, mumps, rubella, tetanus, and poliomyelitis. Low mutation rates due to constraints, such as limited host range in the case of measles,^1^ are key to ensuring long-term immunity by memory B cell and T cell persistence. Vaccination can lead to immunity for up to 30 years and longer for the hepatitis B virus.^2^ The influenza viruses, especially influenza A, show rapid antigenic drift, necessitating extensive modeling to predict the most likely strains to be targeted by the annual vaccine.^3^ Severe acute respiratory syndrome coronavirus 2 (SARS-CoV-2) also shows high mutation rates, facilitating its immune escape. The COVID-19 pandemic caused by the SARS-CoV-2 virus sparked advancement and innovation in the field of serological testing with regard to both binding and neutralizing antibody detection. The virus consists of four proteins: nucleocapsid (N), envelope (E), membrane (M), and spike (S). Interaction with the host cell is mediated by the S protein as the receptor binding domain (RBD) of the S1 subunit binds to the human angiotensin converting enzyme 2 (ACE2) receptor.^4^ Cellular transmembrane protease serine 2 (TMPRSS2) and lysosomal cathepsin proteases cleave the S1 and S2 subunits, followed by membrane fusion initiated by the S2 subunit.^5^ While some antibodies target the nucleocapsid protein, the majority are directed against the spike protein, more specifically RBD.^6,7^ Most SARS-CoV-2 vaccines make use of this observation by introducing mRNA or viral vectors to induce the expression of the S protein as the antigen^8,9^ or by directly introducing the S protein.^10^ Testing for antinucleocapsid antibodies can thus be used to check for past infections, unless an inactivated vaccine was used, introducing all viral proteins.^11^ Antispike and anti-RBD antibodies, which are produced after both infection and vaccination, provide a means to assert the immune status and might serve as a correlate of protection (CoP).^12−14^ For more information on the definition of a CoP for SARS-CoV-2 and other viruses, the reviews by Perry et al.,^14^ Sobhani et al.,^15^ and Plotkin^16^ are recommended.

Two categories of antibodies can be quantified, binding antibodies and neutralizing antibodies (nAbs). The former includes all antibodies directed against a certain antigen, while the latter includes only antibodies that prevent infection, i.e., by blocking the virus–host interaction or preventing the host–cell fusion. Neutralization tests mimic the interaction of the virus with the host cell and thus quantify the neutralizing antibodies indirectly via their ability to block the interaction. The gold standard is the plaque reduction neutralization test (PRNT), which uses live virus incubated with patient serum dilutions prior to the addition to cells expressing the respective viral receptor. Infection of the cells by the virus results in the formation of plaques, which are quantified by manual or automatic counting. The PRNT_50_ value correlates to the serum dilution required to reduce the plaque formation observed without serum by 50%. The use of live virus makes the PRNT and other conventional neutralization tests (cVNTs), such as the micro neutralization test (microNT), highly accurate but requires a biosafety level (BSL) facility of the virus and results in long turn-around times of up to 3 days.^17^ In the case of the viruses mentioned above, BSL-3 would be required for most. To reduce the safety requirements to at least BSL-2, pseudovirus-neutralization tests (pVNTs) have been developed, relying on the use of lentiviruses or vesicular stomatitis virus pseudotyped with the respective viral protein responsible for host cell binding and fusion.^18−20^ For more information on live virus neutralization test and pVNT development, the recent reviews of Rocha et al.,^21^ Sun et al.,^22^ and Vaidya^23^ are recommended. In the case of SARS-CoV-2, RBD was identified as the main target for neutralizing antibodies, providing the opportunity to further simplify the neutralization tests. Surrogate virus neutralization tests (sVNTs), the focus of this review, rely on the competitive binding of neutralizing antibodies and the cell receptor with the relevant viral protein. In the case of SARS-CoV-2, this is ACE2 and RBD, respectively. These cell-free assays can be divided into two categories: high-throughput screening (HTS) and point-of-care (POC) assays. They do not require any biosafety facilities and have rapid turn-around times, making serological testing widely available. The COVID pandemic showed that such tests can be applied to monitor the development of antibody titers in vaccination studies, providing insights into the immunity against SARS-CoV-2. Still, such sVNTs are not endorsed by regulatory agencies to monitor the immune status, yet.^24^ To date, only three sVNTs have been granted emergency use authorization (EUA) by the Food and Drug Administration (FDA).^25^ Lacking standardization and validation during test development as well as difficulties defining a neutralizing antibody titer as CoP due to the rapid mutation of the virus currently hinders progress to take full advantage of sVNTs. However, they are the scientific and technological answer to broad serological testing needed and not only in pandemic situations. In the following, an overview of the development in the field of sVNTs of the last three years is provided. Advantages and disadvantages of the different formats are critically analyzed, and future development potential, especially the applicability toward other viruses, is discussed.

## Binding Antibody Tests

**Figure 1:**
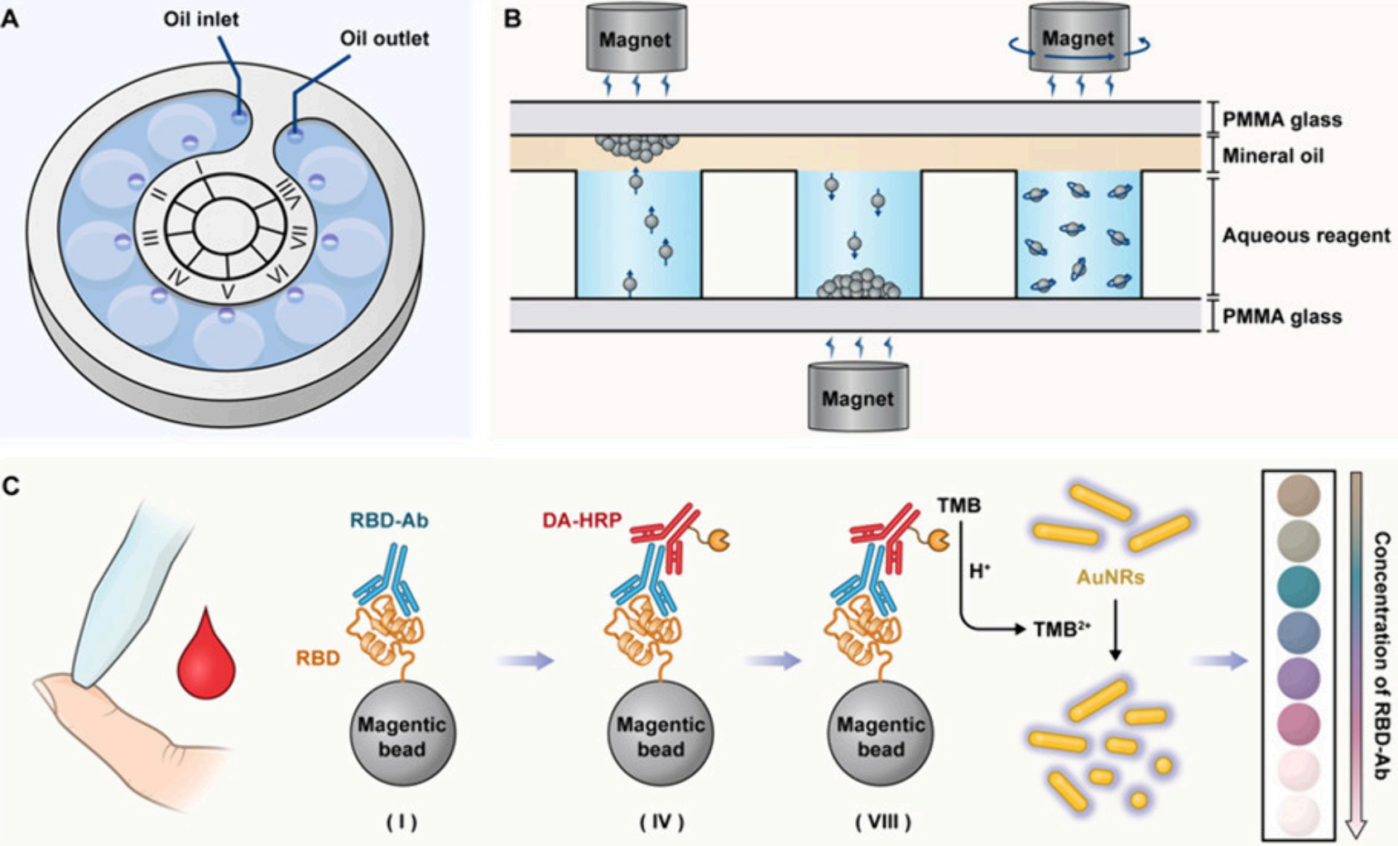
Schematic illustration of the MMI-chip. (A) Structural diagram of the chip with its eight liquid wells for loading sample (I), washing solution (II and III), detection antibody (IV), washing solution (V–VII), and signal substrate (VII). (B) Magnetic bead transportation between wells and mixing using a magnet. (C) Workflow of antibody detection in peripheral blood. Reproduced with permission from ref (31). Copyright 2022 American Chemical Society.

Quantification of patient binding antibodies is used for the assessment of past infections within minutes or hours at the POC. Such tests typically rely on the use of secondary antibodies, which recognize sections of the binding antibody molecules. Thus, such approaches need to account for the different isotypes of patient antibodies and their respective seroconversion. Specifically, immunoglobulin M (IgM) levels rise quickly after infection or vaccination and drop shortly after recovery, while IgA and IgG levels take longer to increase and decrease and are therefore more reliable to serve as indicators for immunity against reinfection.^26,27^ Time-resolved screening with combinations of IgM and IgG binding antibody tests can provide detailed information about the seroconversion after infection or vaccination. Such tests only use selected proteins of the virus and can therefore be used without the need for specialized biosafety facilities. It is important to note that the strength of these tests lies in their ability to provide insights about the immune status of large parts of the population. However, because infectiousness precedes seroconversion by multiple days, binding antibody tests are unsuitable as diagnostic tests to stop the spread of the disease. Still, research for improving their sensitivity and specificity, while maintaining the ease-of-use of the standard rapid lateral flow assay (LFA), has intensified over the last 5 years with some remarkable novelties. For example, Hossain et al.^28^ used alkaline phosphatase (AP)-conjugated secondary antibodies to enable the use of off-the-shelf glucometer test strips by the incorporation of maltose phosphatase. Streptavidin magnetic nanoparticles were modified with biotinylated RBD and incubated with serum and the secondary antibodies. Alkaline phosphatase yellow (pNPP) is enzymatically degraded to PO_4_^3–^, which maltose phosphorylase stoichiometrically converts to glucose, which is then quantified amperometrically in a minipotentiostat. A similar approach was investigated by Peng et al.,^29^ who used AP-conjugated secondary antibodies to enable electrochemical detection of antibodies using a commercial hand-held potentiostat. Their serological testing platform for the rapid electrochemical detection of SARS-CoV-2 antibodies (SPEEDS) consisted of a streptavidin-coated carbon working electrode modified with biotinylated RBD, a carbon counter electrode, and a Ag/AgCl reference electrode. The presence of antibodies was quantitatively determined by the conversion of *p*-aminophenyl phosphate to *p*-aminophenol by AP, which was then oxidized to *p*-quinonimine during chronoamperometry. Other researchers focused on the development of new materials for binding antibody tests. The electrochemical sensor from Nunez et al.^30^ was based on zinc oxide nanorods modified with the S protein, facilitating antibody detection in only 5 min. The positive charge of ZnO makes it an interesting option for the adsorption of negatively charged proteins. Further optimization is needed to make the technology market ready, as the system is only stable for 15 days so far. Nanorods were also involved in Shen et al.’s work, who developed a magnetofluid-integrated multicolor immunochip (MMI-chip).^31^ The eight liquid storage wells of the MMI-chip connected by a mineral oil layer enable multiple reaction and washing steps of magnetic nanoparticles modified with RBD (Figure 1). The horseradish peroxidase (HRP)-labeled secondary antibodies are used to oxidize the substrate 3,3′,5,5′-tetramethylbenzidine (TMB), which then etches gold nanorods, decreasing their length and thereby changing the absorption spectra, enabling a semiquantitative visual read-out. Although being a multistep assay, the chip design results in a sealed environment, minimizing external exposure and increasing user-friendliness. Chip fabrication was also the focus of many other publications. Kim et al. developed a microfluidic fluorescent LFA with integrated dry reagents, a mixer, and a vacuum pump.^32^ The sample first passes DyLight 550-labeled secondary antibodies in a dry reagent storage chamber, is mixed in a herringbone mixer, and then passes the spike-labeled polystyrene particle storage chamber. These particles are then captured at the detection zone due to pillars, enabling fluorescence measurements using an inverted microscope.

**Figure 2:**
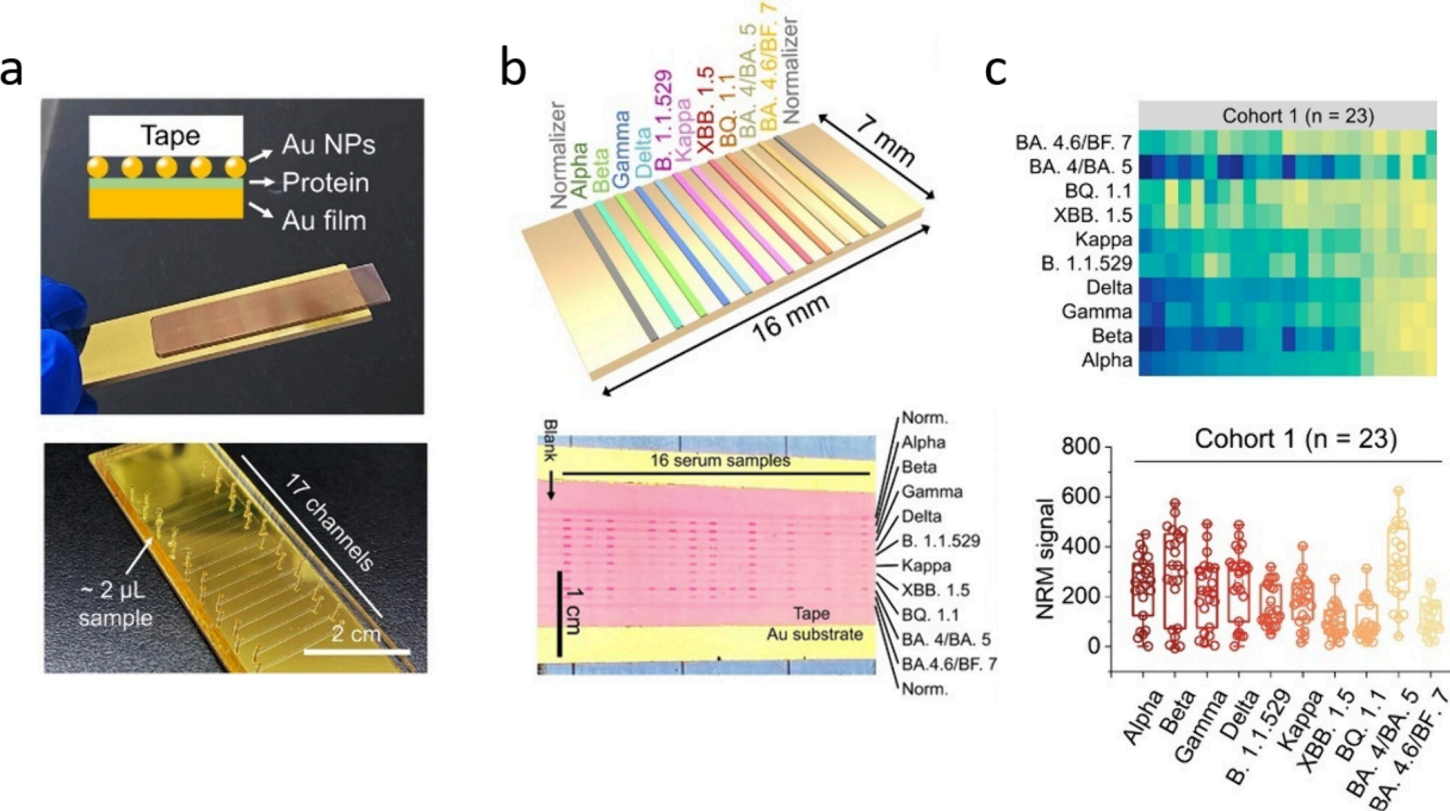
Visualization of the naked-eye readable barcode and micromosaic (NRM) assays. (a) Schematic and photograph illustrating the ready-to-use NRM chip (top) and the microfluidic channels for the serum incubation (bottom). (b) Schematic representation of the antigen immobilization layout of the barcode NRM assay (top). Smartphone image of the micromosaic NRM chip, allowing for simultaneous testing of up to 16 samples (bottom). (c) Heatmap (top) and box plots (bottom) showing levels of antibodies against multiple SARS-CoV-2 variants in serum samples from random donors after the reopening in China in December 2022 (Cohort 1). Reproduced with permission from ref (35). Copyright 2023 American Chemical Society.

Multiplexing offers another intriguing avenue, allowing for simultaneous IgG and IgM detection of a variety of antigens, opening the possibility to screen several viral variants or several prevalent viruses simultaneously. In the case of SARS-CoV-2, this is of growing interest, as it continuously mutates so that the dominating variant changes quickly. Several multiplexing platforms were developed relying on machine learning, magnetic barcode beads, nanopore sensing, and more.^33−37^ The paper-based multiplexing vertical flow assay (xVFA) by Eryilmaz et al. was able to screen five different SARS-CoV-2 antigens for IgG and IgM antibodies in <20 min.^33^ Their device was 3D-printed and used a mobile-phone-based optical reader. The neural network showed 89.5% accuracy for 31 serum samples tested after training. Importantly, they also addressed the limitations caused by the choice of serum panel used during development versus later application. Serum panels consisting of local sera or small numbers of sera may be biased toward less variations in vaccination and infection status. Nan et al.’s naked-eye readable microarray (NRM) based on a thickness sensing nanoplasmonic ruler provided a rapid and POC friendly multiplex sensing platform (Figure 2).^35^ Their NRM chips can screen 10 different RBD variants for 16 serum samples (∼2 μL each) simultaneously in <30 min. The test is based on a gold nanoparticle (AuNP) monolayer, where the increased thickness caused by captured antibodies results in a decreased reflectance, which, in turn, is measurable using gray value analysis of smartphone images. Their manufacturing requires a tape-based transfer of the AuNP monolayer, which is deemed impractical, however. Exploiting similar effects, Huang et al. have developed a nanoplasmonic immunosensor platform using nanoporous hollow gold nanoparticles modified with RBD to detect anti-RBD antibodies on an anti-IgG coated nanoplasmonic sensor chip.^38^ The system can generate a signal within 15 min without the need of signal amplification or washing. Similarly, dual-affinity ratiometric quenching (DARQ) can enable a fast signal generation in a homogeneous format. Kilgour et al. mixed serum with fluorescein-labeled RBD and rhodamine-labeled protein L, obtaining signals in just over 2 min.^39^ Liang et al. combined a visual LFA with Raman spectroscopy.^40^ They synthesized silver nanoparticles with ultrathin gold shells embedded with 4-mercaptobenzoic acid and conjugated them with secondary antibodies using HS-PEG-COOH and EDC/NHS chemistry. The particles enabled dual-mode qualitative visual and quantitative SERS read-out via a portable Raman spectrometer with a 785 nm laser on a LFA test strip. They later changed to a competitive assay format to allow the detection of neutralizing instead of binding antibodies.^41^ This is an excellent example demonstrating that the noncompetitive format of binding antibody tests can often be changed to a competitive format, making the technologies even more universally applicable. In fact, many of the sVNTs discussed in the next chapters have evolved out of binding antibody tests. More information on binding antibody tests can be found in the reviews written by Lee et al.,^42^ Yari et al.,^43^ and Dong et al.^44^

## Commercial Binding and Neutralizing Antibody Tests

**Figure 3:**
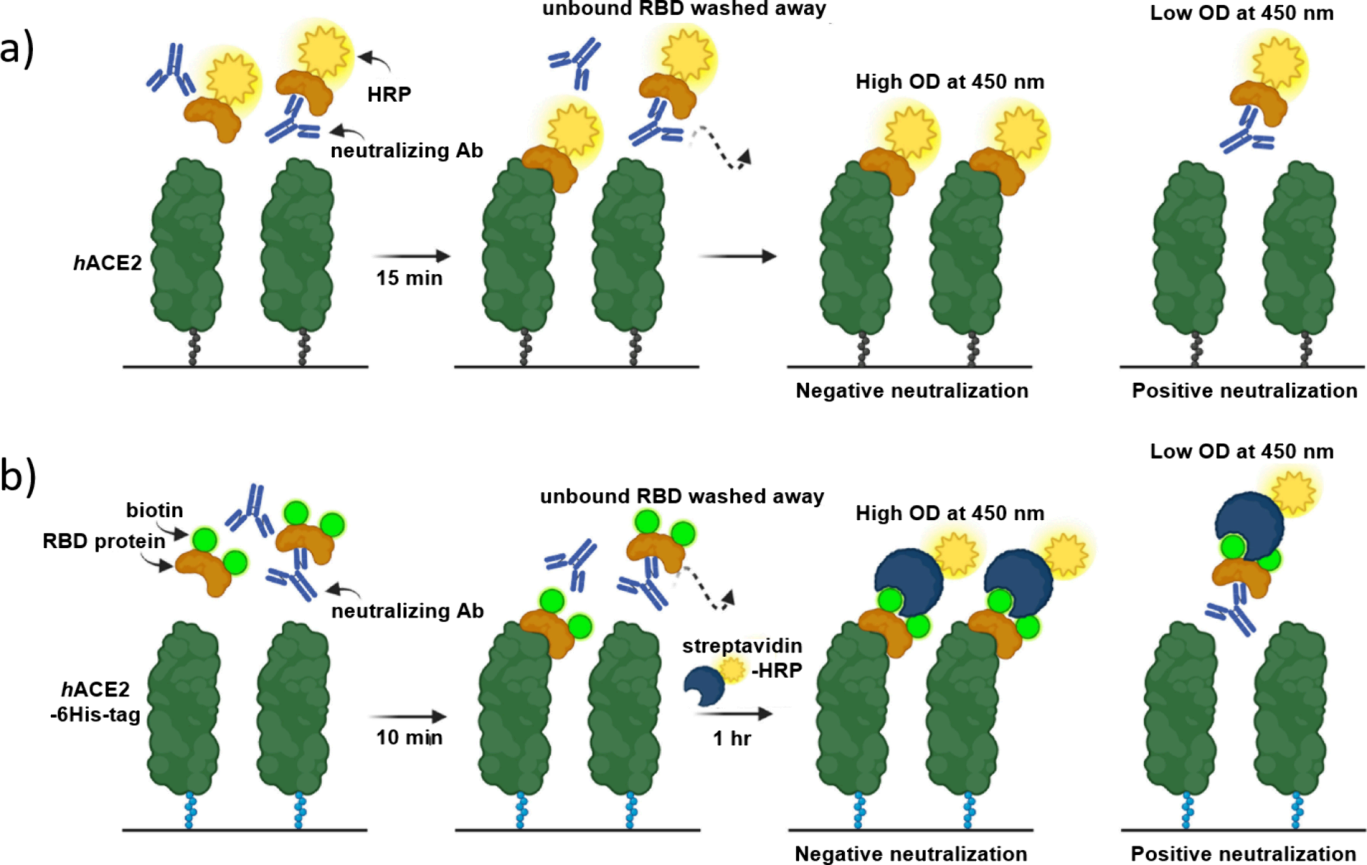
Illustration of the assay principle of (a) GenScript’s cPass sVNT kit and (b) Ahn et al.’s biotin-based sVNT. Reprinted with permission from ref (62). Copyright 2023 Elsevier.

The cell-free detection of antibodies directed against viral diseases has long been established, starting with enzyme-linked immunosorbent assay (ELISA) concepts against measles, rubella, and hepatitis^45−47^ and, more recently, the development of LFAs.^48^ In the case of the COVID-19 pandemic, large companies quickly adapted existing technologies to provide diagnostic tools for SARS-CoV-2, including antibody detection. Many of the resulting products gained EUA by the FDA, as indicated above, to enable their distribution, without the need to go through the complete FDA authorization process. Now, over 5 years after the beginning of the outbreak, some of these EUAs have been revoked,^49^ and most products are off the market. Once discontinued, finding detailed information about the testing principles behind the products can be difficult, as datasheets become unavailable. In these cases, publications using the tests or Web sites listing multiple tests are the best option to obtain more information. At the point of writing, the FDA still lists 75 serological tests granted EUA, the first one issued April 2020 and the most recent one in July 2024^25^ and two granted traditional marketing authorization.^50^ Of these, most detect IgG, IgM, or total antibody levels. Only three are targeting neutralizing antibodies: the SCoV-2 Detect Neutralizing Ab ELISA (InBios International, Inc.), the Diazyme SARS-CoV-2 Neutralizing Antibody CLIA Kit (Diazyme Laboratories, Inc.), and the cPass SARS-CoV-2 Neutralization Antibody Detection Kit (GenScript). The list reveals that, besides the classification into binding and neutralizing antibody tests, there are two major groups of tests: ELISAs and chemiluminescence immunoassays (CLIAs) for high-throughput screening and LFAs for the POCT. The former are mainly microtiter plate assays or microarrays using secondary antibodies labeled with HRP, fluorescence, or chemiluminescence markers. The predominant commercial sVNT in literature is GenScript’s cPass SARS-CoV-2 Neutralization Antibody Detection Kit, which uses RBD-conjugated HRP and an ACE2-coated 96-well microplate (Figure 3a).^51−54^ Two separate incubation steps at 37 °C are required before signal generation through TMB, with an overall assay time of ∼1 h. Many similar assays have been on the market, varying mainly in incubation times; examples are the Leinco COVID-19 ImmunoRank (Leinco Technologies, Inc.)^53^ and TECO SARS-CoV-2-AK Surrogate Neutralisation Test (TECOmedical).^51,53^ Fluorescence or chemiluminescence read-outs can shorten the assay time and reduce assay steps and are often fully automated systems. Examples for chemiluminescence assays are the Roche Elecsys Anti-SARS-CoV-2 S,^55^ which uses biotinylated RBD, ruthenium–RBD conjugates, and streptavidin-conjugated particles for binding antibody detection, and the Diazyme SARS-CoV-2 Neutralizing Antibody CLIA Kit, relying on RBD-modified magnetic microbeads and ACE2-ABEI. They are automated and can generate signals in 18 and 34 min, respectively. Graninger et al. compared seven commercial immunoassays and found that the cPass SARS-CoV-2 Neutralization Antibody Detection Kit showed the most robust quantitative correlation with a live-virus neutralization test, while the ACE2-RBD neutralization assay by DiaPro and the TECO SARS-CoV-2 neutralization antibody assay displayed the highest sensitivity for nAb detection.^51^ For the POC detection, LFAs relying on AuNPs or fluorescent markers for signal generation reached the market. The Healgen Scientific SARS-CoV-2 Neutralizing Antibody Rapid Test Cassette^55,56^ comprises RBD-conjugated AuNPs, which are captured on the ACE2 test line in the absence of neutralizing antibodies. A portable reflectance spectrum analyzer can be used to quantify the response. Similarly, the VERI-Q SARS-CoV-2 Neutralizing Antibody Rapid Test Kit (MiCo BioMed)^57^ captures AuNP-RBD bound to ACE2-mouse-Fc on a goat antimouse IgG test line, requiring only 10 μL of serum. More sensitive results can be obtained with fluorescent RBD conjugates used for example in the ichroma COVID-19 nAb test.^58^ It is based on capturing an ACE2–biotin conjugate on a streptavidin test line, making it susceptible to interference by biotin. Users are therefore advised to use the test only 24 h after stopping the intake of biotin supplements to ensure assay functionality, where concentrations of 500 ng/mL were shown not to interfere with the assay. When the 1:5 dilution of serum is factored in, this is only slightly below the threshold of 3510 ng/mL (14367 nM) mentioned in the Clinical and Laboratory Standards Institute (CLSI) guideline (EP37), which is 3 times the highest physiological biotin concentration measured in a patient with high biotin dose uptake.^59^ Actually, this is a common problem for diagnostic tests using the streptavidin–biotin interaction, thus testing for biotin interference should be kept in mind during assay development. A different approach is pursued by the PremaLabs Diagnostics SARS-CoV-2 NAb test kit. According to McLean et al., it uses an anti-RBD test line to capture neutralized fluorescently labeled RBD.^60^ In the absence of neutralizing antibodies, RBD is bound by ACE2, blocking the binding site of the capture antibody. This approach appears problematic, as neutralizing antibodies binding in a position similar to that of ACE2 could in theory also prevent capture at the test line, causing a false negative result. An advantage of the assay is that it also works with whole blood, while many other tests require serum or plasma. The fluorescence LFIA Finecare 2019-nCoV S-RBD test was advertised with a short assay time of 15 min.^61^

To compete with commercially available products, newly developed sVNTs should factor in several characteristics. (I) The assay type, i.e., HTS or POC format, including consideration for the involved steps, turn-around time, and need for specialized equipment, which are directly interconnected with the cost of the test. (II) The type of sample that can be used, i.e., serum, plasma, or preferably (finger prick) whole blood for the POC. (III) Desired sensitivity, i.e., qualitative, semiquantitative, or quantitative read-out. Besides the choice of signaling agent, the sample volume is important because it directly affects sensitivity. (IV) The choice of conjugation strategy needs to account for the option of adaptation to other variants or viruses. This is intertwined with the protein expression and purification strategy.

## New Concepts for sVNTs

The following chapters will discuss the advantages and disadvantages of new sVNTs, taking into account their applicability as a possible commercial product. For a better overview, they are grouped into HTS and POC formats, with subchapters concerning the method of signal generation and the special cases of homogeneous HTS and signal-on POC sVNTs.

## High-Throughput sVNTs

### HRP-Based Detection

Many variations of HRP-based sVNTs have been published, changing target and capture protein as well as signaling conjugates. Correlations to either PRNT or pVNT were provided for some but not all of these, complicating comparison of the formats. Table 1 lists the capture protein, target protein, signaling conjugate, substrate, incubation time, and steps as well as correlations to other neutralization tests for 15 HRP-based sVNTs for easier comparison. Ahn et al. immobilized ACE2 via His_6_-tag for improved binding of RBD (Figure 3b) compared to the commercial cPass sVNT from GenScript (Figure 3a).^62^ They biotinylated RBD using sulfo-NHS-biotin, which is a simple but random strategy because not only the N-terminus but also lysine residues can be biotinylated. Due to a washing step before addition of the streptavidin–HRP conjugate, no interference of biotin is to be expected. The developed sVNT correlated well with both a pVNT (*R*^2^ = 0.9006) and the cPass sVNT (*R*^2^ = 0.8521), as investigated with a panel consisting of 100 sera, suggesting that biotinylation did not affect antibody or ACE2 binding. Only 2 ng of RBD biotin are needed per well, while other assays use 100 ng of RBD for coating.^63^ This resulted in improved sensitivity, allowing for the use of lower sample volumes compared to its commercial counterpart. Biotinylation of RBD for site-directed immobilization in neutravidin or streptavidin plates should also allow for reduced amounts of RBD in the system.^64,65^ Kolesov et al. compared the ACE2 plate plus RBD-HRP system to the RBD plate plus ACE2-HRP system and found the latter to be superior due to better storage performance and easier adaptation to other variants.^66^ Mutation of SARS-CoV-2 might affect conjugation, for example, by causing different biotinylation patterns for RBD variants as lysines might be exchanged or their direct environment altered. However, this could also affect the immobilization of the protein in the plate. The introduction of Cys-tags or Avi-tags for directed conjugation could be a solution but is time-consuming and might affect protein folding. Liu et al. bypassed such problems by designing recombinant ACE2-Fc-Avi and RBD-Fc-vHRP proteins.^67^ The IgG Fc fragment facilitated both purification and dimerization of the ACE2 fusion protein, improving its affinity to RBD. Avi-tag allowed for biotinylation and thus site-directed immobilization in a streptavidin plate. Furthermore, RBD-Fc-vHRP showed ∼7 times higher affinity to ACE2 compared to RBD-vHRP. They state that the approach allows for easy adaptation for other RBD variants because it eliminates the purification and conjugation step for the protein.

**Table 1:** List of HRP-Based sVNTs

capture protein (immobilized)	target protein	signaling conjugate	substrate	correlation to other neutralization tests	incubation steps and time (excl. substrate reaction)	ref
ELISAs						
(Common Microplate-Based Assays)						
His_6_-ACE2	RBD-biotin	streptavidin-HRP	TMB	pVNT, *R*^2^ = 0.9006, *n* = 100	10 min + 1 h RT	(62)
				cPass, *R*^2^ = 0.8521, *n* = 100		
ACE2	His-tag-RBD	anti-His-Ab-HRP	TMB	pVNT, *r*^2^ = 0.7135, *n* = 62	2 × 1 h 37 °C	(72)
ACE2	StrepTag-Spike	anti-Strep-Ab + anti-Fc-HRP	TMB	cPass, *R*^2^ = 0.9129, *n* = 32	1 h 37 °C + 1 h RT	(73)
ACE2-Fc-Avi-biotin (streptavidin plate)	RBD-Fc-vHRP	RBD-Fc-vHRP	TMB	pVNT, *R*^2^= 0.91, *n* = 19 (WT)	2 × 1 h 37 °C	(67)
				pVNT, *R*^2^ = 0.90, *n* = 15 (delta)		
ACE2/RBD	RBD/ACE2-HRP	RBD/ACE2-HRP	TMB	PRNT, *r* = 0.855, *n* = 73	2 × 30 min 37 °C	(66)
RBD	ACE2-biotin	poly-HRP-streptavidin	TMB	PRNT, *R*^2^ = 0.6, *n* = 57	2 × 1 h RT	(63)
				pVNT, *R*^2^ = 0.76, *n* = 20		
RBD	ACE2-3xFLAG	anti-FLAG-Ab-HRP	TMB	cVNT, *r*^2^ = 0.97, *n* = 26 (nonlinear fit)	3 × 30 min 37 °C	(74)
				pVNT, *r*^2^ = 0.90, *n* = 29 (nonlinear fit)		
RBD	ACE2-biotin	streptavidin-HRP	SigmaFast OPD	only five samples compared to pVNT	2 × 1 h + 20 min RT	(75)
mFc-RBD	ACE2-biotin	streptavidin-HRP	TMB	PRNT, *r*_S_ = 0.83, *n* = 32	30 min + 2 × 1 h RT	(76)
RBD-biotin (neutravidin plate)	ACE2-Fc	protein-l-HRP	TMB	pVNT, *r* = 0.74, *n* = 144	1 h + 30 min RT	(64)
RBD-biotin (ELISA plate)	His-tag-ACE2	anti-His-Ab-HRP	TMB	not correlated	2 h + 1 h RT	(65)
ACE2	spike-biotin	streptavidin-HRP	CL substrate	only used for antibody screening	1 h + 20 min +4.5 min	(77)
						
Alternative Formats Using HRP Conjugates for Signal Generation						
(Microarray, Fiber-Optic Biolayer Interferometry, Track-Etched Microporous Membrane)						
ACE2	RBD-biotin	streptavidin-HRP	H_2_O_2_/luminol	YHLO NT, *R* = −0.87, *n* = 33	7 min	(68)
ACE2-biotin (streptavidin probe)	RBD-HRP	RBD-HRP	DAB	ELISA, *r* = 0.859, *n* = 15	5 min RT (+2 min substrate)	(69)
			AMEC	pVNT, *r* = 0.983		(70)
				three sera tested for three variants		
ACE2-PS-microbeads (in solution)	RBD-HRP	RBD-HRP	TMB	pVNT, *R*^2^ = 0.7856, *n* = 81	30 min RT	(71)

Aside from conjugation strategies, the assay layout is of consideration. ELISAs are typically performed in 96-well plates, requiring relatively long incubation times and multiple washing steps. Klüpfel et al. have developed a microarray with chemiluminescence read-out using streptavidin-HRP and H_2_O_2_/luminol to obtain signals in only 7 min.^68^ Similarly fast was the approach of Bian et al., who used fiber-optic biolayer interferometry in a 96-well plate. The fiber-optic probe is coated with streptavidin and biotinylated ACE2 to capture RBD-HRP. They started using 3,3′-diaminobenzidine tetrahydrochloride (DAB), a metal precipitating substrate for signal generation,^69^ but later found a more environment- and user-friendly biomaterial, 3-amino-9-ethylcarbazole (AMEC), that increased signal-to-noise ratios and enabled fiber regeneration up to 6 times.^70^ The later version was also used for multiplexing. Wang et al. developed another device using fiber optics.^71^ Their track-etched microporous membrane filtration microplate allows for washing via capillary siphoning, solely requiring absorbent paper. Read-out via smartphone is possible due to the use of individual optical fibers connected to the 64 wells, making the device usable at the POC.

### Fluorescence Detection

**Figure 4:**
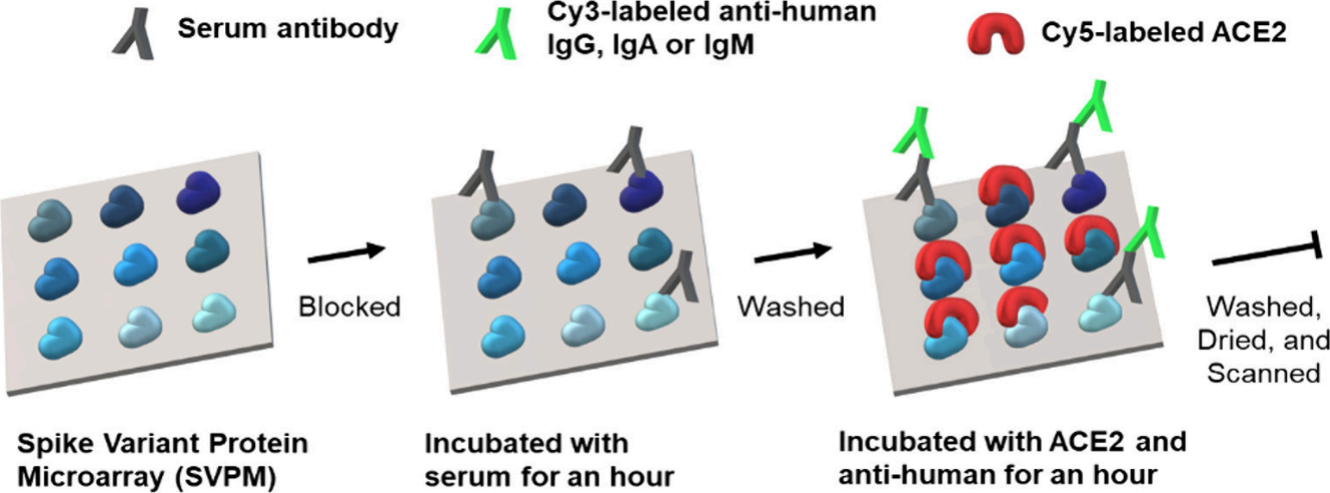
Schematic of the assay procedure for the spike variant protein microarray. Reproduced with permission from ref (88). Copyright 2022 American Chemical Society.

Fluorescence detection for sVNTs to improve sensitivity compared to the HRP-based assays or to enable multiplexing was investigated by multiple groups. Table 2 lists the capture protein, target protein, signaling conjugate, substrate, incubation time, and steps as well as correlations to other neutralization tests and comments for 10 fluorescence-based sVNTs for easier comparison. Most dominant is the use of phycoerythrin-conjugated streptavidin as the signaling conjugate.^78−83^ Combined with beads that are labeled with a set of spectrally distinct dyes, the main advantage of this approach is its potential for multiplexing. When scanned individually with a red 635 nm laser in a flow cell, up to 80 different beads can be classified and analyzed by the excitation of phycoerythrin with a green 532 nm laser using a Luminex instrument.^78^ MagPlex or other magnetic beads were labeled with ACE2, Spike, or RBD as the capture probe, incubated with the target (Spike-biotin or ACE2-Fc/biotin) and serum, followed by the signaling conjugate before analysis in the respective device. The studies revealed good correlation of the developed sVNTs to microneutralization tests, PRNT or cVNT, highlighting their sensitivity. The automation of the system allowed for the screening of larger serum panels, with several hundred being used for validation in some cases. Hoffman et al.^80^ and Lynch et al.^81^ made use of the multiplexing capability and tested several variants. Their assay also had the lowest turnaround times with 60 and 52 min, respectively. Unfortunately, the correlation to different established neutralization tests by each group makes a direct comparison between these sVNTs impossible. This highlights a major obstacle with neutralization tests in general and a call for widely available standards. The WHO generated the international standard for anti-SARS-CoV-2 (NIBSC code: 20/136) and an international reference panel for anti-SARS-CoV-2 (NIBSC code: 20/268) consisting of a seronegative and four seropositive samples with low-to-high antibody levels. Demand obviously was greater than availability, and hence these were only obtained by some of the interested industrial and academic parties. Aside from Hoffman et al.,^80^ the reference panel was also used by Ho et al.,^84^ who developed a microarray with Spike variant dots and Cy5-ACE2 and Cy3-antihuman antibodies for signal generation. Their test, referred to as CoVariant, showed an excellent correlation to GenScript’s cPass sVNT for the WHO reference panel (*R*^2^ = 0.9728) and was used for the alpha, beta, gamma, delta, and omicron (B.1.1.529) variants in clinical follow-up studies.^85,86^ Results for a similar assay referred to as CoVariant-SCAN were previously published by Heggestad et al., who used RBD instead of Spike and AlexaFluor647-labeled ACE2 instead of Cy5.^87^ Another Spike microarray was developed by Su et al. for antibody profiling and ACE2 inhibitor screening using ACE2-biotin and Cy5-streptavidin and Cy3-anti IgG/A/M (Figure 4).^88^ Yang et al. devised an automatic testing-on-a-probe biosensor.^89^ The RBD-modified probe was subsequently incubated with biotinylated ACE2 and Cy5-streptavidin-polysaccharide before read-out with interim wash steps. This generated qualitative results within 18 min for a single sample or 40 min for 20 samples and correlated well to both PRNT and pVNT. A different advantage can be gained by using liposomes encapsulating sulforhodamine B (SRB), as they facilitate both fluorescence and colorimetric read-out and can thus be used for both an ELISA-type HTS and an LFA-based POC assay,^90^ respectively.

**Table 2:** List of Fluorescence-Based sVNTs

capture protein	target protein	signaling conjugate	comments and correlation to other neutralization tests	incubation steps and time	ref
Luminex Platform					
Spike-MagPlex beads	ACE2-Fc	antimouse-IgG-phycoerythrin	BioPlex 200 reader (BioRad)	2 × 1 h + 45 min	(78)
			PRNT, *R*^2^ = 0.825, *n* = 206		
RBD/Spike-MagPlex beads	ACE2-biotin	streptavidin-phycoerythrin	MagPix (Luminex)	45 min + 15 min	(80)
			Wuhan, δ, and ο (B.1.1.529) cVNTs (100% qualitative agreement, *n* = 72 + ECDC and WHO standard)		
spike variant magnetic beads	ACE2-biotin	streptavidin-phycoerythrin	BioPlex 2200 (BioRad)	52 min	(81)
			PRNT_50_, *r*_S_ = 0.80 (76 seropositive and 102 seronegative samples), WT, α, β, γ, δ, κ, ε (451 seropositive samples)		
ACE2-MagPlex beads	Spike-biotin	streptavidin-R-phycoerythrin	FlexMap 3D (Luminex) microNT (several hundred sera, no statistical analysis)	2 × 1 h RT	(82)
					
Microarrays					
spike variants	Cy5-ACE2	Cy3-antihuman-Ab	WHO reference panel in cPass and CoVariant (*R*^2^ = 0.9728, *n* = 5)	2 × 1 h	(84)
			simultaneous detection of Ig possible		(85)
					(86)
RBD variants	ACE2-AlexaFluor647	ACE2-AlexaFluor647	CoVariant SCAN (WT, B.1.1.7, B.1.351, and P.1)	1 h	(87)
			microNT, *r* = 0.74, *n* = 16		
spike variants	ACE2-biotin	Cy5-streptavidin	focus on antibody profiling	2 × 1 h	(88)
		Cy3-anti IgG/A/M	ACE2 inhibitors ramipril and perindopril		
					
Others					
RBD-coated probe tip	ACE2-biotin	streptavidin-Cy5-polysaccharide	automated system	40 min (20 tests)	(89)
			PRNT, *r* = −0.82, *n* = 46	18 min (1 test)	
			pVNT, *r* = −0.80, *n* = 46		
ACE2	RBD liposomes encapsulating SRB	RBD liposomes encapsulating SRB	dual use for colorimetric LFA and fluorescence microplate HTS assay	1 h 30 °C + 2 h RT	(90)
			pVNT, *r*_S_ = 0.847, *n* = 20		
ACE2-biotin-SA beads	RBD/S1-Fc	AF488-conjugated polyclonal secondary Ab or PE-anti His tag monoclonal Ab	flow cytometry, investigation of different models	30 min 37 °C	(91)
S1-biotin-SA beads	ACE2-Fc/His		iACE2/RBD-Fc, iACE2/S1-Fc, and iS1/ACE2-His worked best	30 min + 20 min	
RBD-biotin-SA beads	ACE2-Fc/His		PRNT, 3R = 0.896, *n* = 20 (iACE2/RBD-Fc)	RT	

### Other Detection Methods

In addition to the two major detection categories of HRP or fluorescence-based detection strategy, SPR, thin-film interferometry, bioluminescence, and chromogenic read-outs have been described, which may provide additional information through their different signaling mechanisms.^92−96^ Dong et al. tested online functionalization of a four-channel SPR chip with S1, Protein G, and ACE2 for the simultaneous quantification of antibodies and fully and partially neutralized viral particles.^92^ The value of this approach is provided through its detailed information for antibody screening with a reasonable sample throughput considering key performance characteristics of a turn-around time of <12 min and autosampler-enabled continuous measurements with a single chip for up to 6 days. Modification of a plasmonic chip with a lipid bilayer as the artificial cell membrane provided a biomimetic nanoplasmonic sensor.^97^ While the test uses the same underlying principle as the other discussed tests and could be used for nAb detection, the authors went instead for the investigation of monoclonal nAbs as an antiviral therapy, highlighting an alternative application of the sVNT platforms. Thin-film interferometry enabled the development of an automated label-free sVNT based on measuring the binding ability of RBD to ACE2 after neutralization.^94^ In a first cycle, the RBD-coated sensing probe is incubated with serum dilutions followed by ACE2 addition after a washing step. In a second cycle, the probe is only incubated with ACE2. The neutralization rate can be calculated as the ratio of the first to the second cycle. Similar to the fluorescence- or HRP-based microarrays discussed above, Springer et al. used a commercial SARS-CoV-2 VoC ViraChip IgG microarray adapted to nAb detection for multiple RBD variants with an ACE2–alkaline phosphatase conjugate for colorimetric signal generation.^96^ A colorimetric read-out was also generated by Kwak et al.’s Janus nanozymes (Ir–Au nanodisks) in combination with TMB/H_2_O_2_.^93^ Recently, the use of nano luciferase (NanoLuc) has come into focus for signal generation. Schoefbanker et al. used it for a sensitive ELISA-type assay, incubating RBD-NanoLuc first with serum followed by incubation in an ACE2-coated plate.^95^ The main advantage, however, lies in the use of split NanoLuc to facilitate homogeneous assay formats that will be discussed in detail in the next chapter.

### Homogeneous Assays

**Figure 5:**
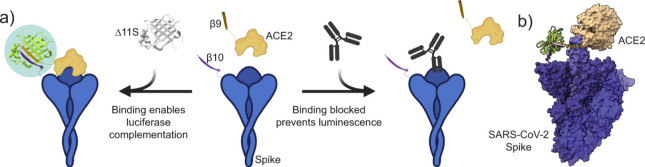
Illustration of (a) the homogeneous sVNT using Tripart NanoLuc peptide fragments and (b) the molecular model of the predicted refolding of NanoLuc (PDB 5IBO) (green) after complementation of fragments β10 and β9 driven by the interaction between SARS-CoV-2 spike trimer (purple) and ACE2 (fawn) (PDB 7A97). Reprinted with permission from ref (100). Copyright 2022 Macmillan Publishers Ltd. The article is licensed under a Creative Commons Attribution 4.0 International License http://creativecommons.org/licenses/by/4.0/.

Heterogeneous sVNTs discussed so far almost all required multiple incubation, addition, or washing steps, naturally, making their manual execution laborious and often time-consuming. This could be overcome in several cases by automation, which usually requires special equipment and is therefore costly. Instead, the concept of split reporter molecules avoids the need for washing steps and in some cases even reduces the number of incubation steps. These include split-NanoLuc,^98−102^ split-oligonucleotides,^103^ and NIR-II FRET systems.^104^ The NanoLuc Binary Technology (BiT) uses two subunits of NanoLuc, the large subunit (LgBiT) consisting of domains β1–9 and the small subunit (SmBiT) consisting of domain β10. The subunits can be either conjugated to proteins or expressed as recombinant fusion proteins, omitting the need for conjugation. The subunits fuse when brought into close proximity, resulting in an active NanoLuc. Alves et al. used commercially available Lumit antibodies (antirabbit Ab-SmBiT and antimouse Ab-LgBiT) and prepared rabbit-Fc-RBD and mouse-Fc-ACE2 for quick adaptation to SARS-CoV-2.^98^ Another group prepared LgBiT-ACE2 and SmBiT-S1, circumventing the additional interaction of secondary antibody and Fc fragment.^99^ Kim et al. went one step further and used the trimeric full-length Spike to detect neutralizing antibodies against the whole protein and not just those directed against RBD or S1.^100^ This remains a topic of debate because many sVNTs rely on the use of only RBD and still correlate well with PRNTs and pVNTs using the whole virus or the S protein. They used a trisplit NanoLuc and produced multiple SmBiT-S variants, including omicron (B.1.1.529), proving the potential for quick adaptation to emerging variants (Figure 5). All assays required 2–3 incubation steps with assay times between 2 h down to slightly over 30 min.^99^ The NanoLuc system has been also otherwise identified as a powerful tool in HTS formats, such as for the detection of binding antibodies against the glycoprotein of the nipah virus, producing comparable results to an ELISA.^105^ The split-oligonucleotide neighboring inhibition assay (SONIA) used real-time qPCR to measure the ability of neutralizing antibodies blocking the binding between DNA-barcoded S1 and ACE2.^103^ Upon interaction of S1 and ACE2, ligation-mediated PCR-amplification of the DNA barcodes is enabled. Due to the amplification reaction, high sensitivity and specificity is achieved in comparison to traditional pVNTs but requires 140 min until read-out. The low volume requirements (4 μL) and broad allowance for the sample type, including serum and dried blood spot eluent, renders it an interesting lab-based technology. Zhao et al. could use whole blood as a sample for their assay, relying on a pair of lanthanide downshifting nanoparticles to enable a near-infrared II Förster resonance energy transfer (NIR-II FRET).^104^ The Nd^3+^-doped particles served as an energy donor and were modified with RBD, while the Yb^3+^-doped ones served as an energy acceptor modified with ACE2. While an interesting concept, the system currently only measures in a cuvette, making it impractical for HTS. Impedance measurements are another possibility to facilitate homogeneous assays, as shown by Manshadi et al. with their ACE2-modified interdigitated electrode, but have only been tested in spiked mouse serum so far.^106^

## POC Neutralization Tests

### Colorimetric and Fluorescence Detection

**Figure 6:**
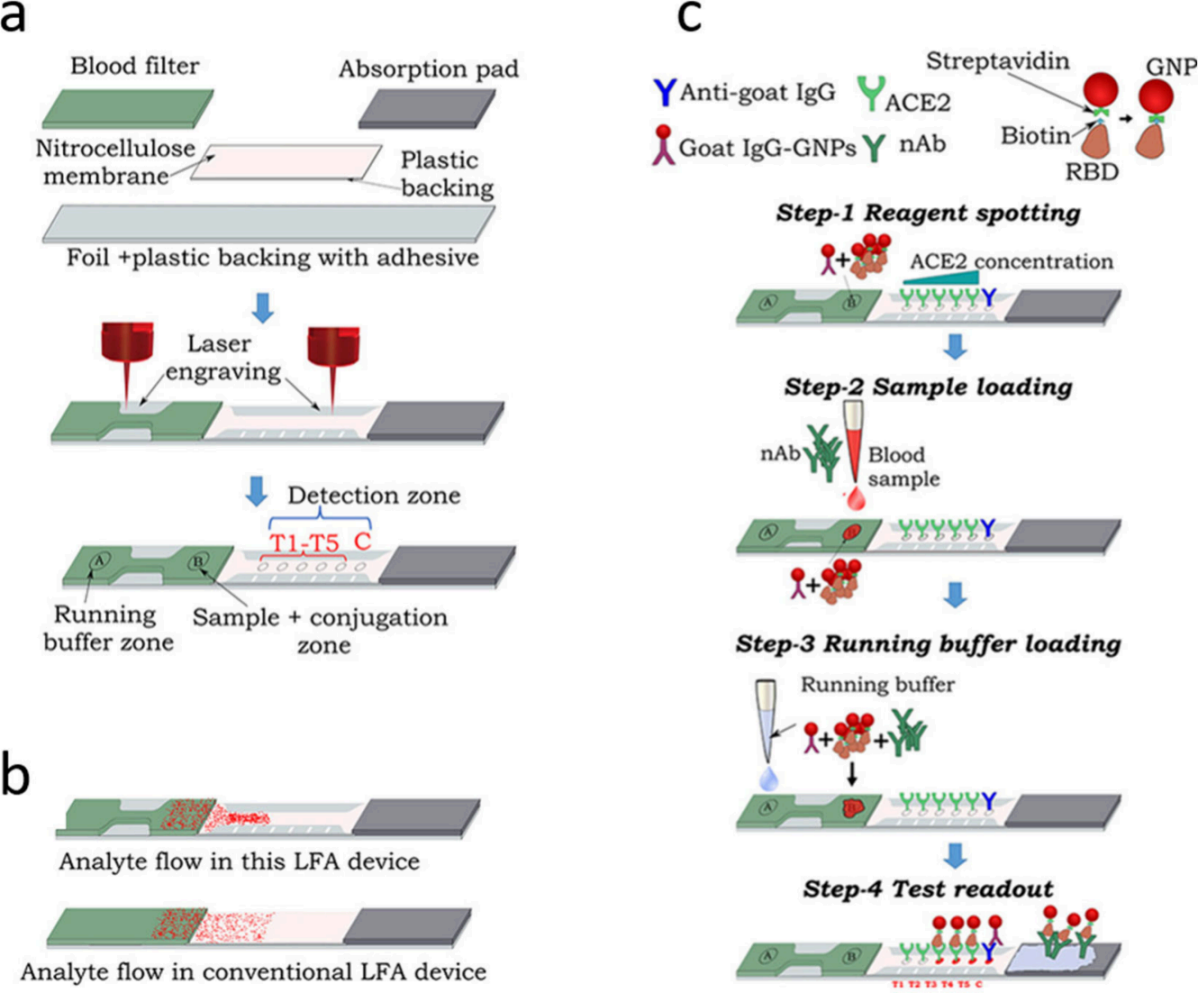
Schematics of the laser-engraved LFNT. (a) Schematic illustrating the fabrication of the LFA test strip. (b) Visualization of the analyte concentration with custom geometry versus traditional LFA geometry. (c) Schematic of the LFNT procedure. Reproduced with permission from ref (108). Copyright 2024 American Chemical Society.

AuNPs, the predominant signaling agent for LFAs, were used in many commercial POC neutralization tests; meanwhile, academia investigated ways to improve these assays and find potential alternatives. A comparison of 11 colorimetric and 4 fluorescence lateral flow neutralization tests (LFNTs) is given in Table 3. Novelties included the overlaying of test strips for multiplexing,^107^ laser cut test strips for improved sensitivity,^108^ and the use of additional test lines.^109^ Specifically, Deenin et al. used a paper puncher to prepare mirrored concave holes on the ACE2 test line of two test strips.^107^ Delta and omicron RBD-AuNPs were immobilized on the conjugate pads, enabling multiplexed read-out of the stacked test strips. The smaller test line additionally resulted in improved sensitivity due to concentration of the nanoparticles on the test line. This was also the reasoning behind Mahmud et al.’s laser engraving of both blood filter and nitrocellulose membrane (Figure 6).^108^ The blood sample is incubated with the RBD-biotin–streptavidin-AuNPs on the filter pad for 2 min before the addition of running buffer. The laser engraved narrow partition slows the flow and concentrates the sample which passes five individual ACE2 spots. The additional spots improve sensitivity and facilitate a semiquantitative read-out by counting of the visible spots. The usability of whole blood makes the assay highly useful for the POC and was therefore investigated by several other groups as well.^109,110^ The addition of an RBD test line by Fulford et al. enabled simultaneous detection of total anti-RBD antibodies.^109^ These correlated less well to a microNT than the neutralizing antibody titers as anticipated, making the added value of such a test line questionable. Variation of the geometric design of the LFA test strips on a large scale for industrial fabrication poses a challenge but could be worthwhile in light of the added benefits. A different solution was presented by a paper-based cellulose-pulldown assay using HRP and TMB for signal generation.^111^ While nitrocellulose is better suited for general immobilization of proteins cellulose is cheaper and more readily available. The use of the cellulose binding domain (CBD) facilitates immobilization of the RBD–CBD fusion protein. Wax printing provides a simple means to generate the desired geometries without the need for laser cutting or similar approaches but has waned in research use recently due to the disappearance of commercially available desktop printers. Other colorimetric LFNTs included the use of nanoshells,^112^ cellulose nanobeads,^113^ red latex microbeads,^114^ and liposomes.^90^ All assays required 10–20 μL of sample, run times varied between 9 and 25 min, and the obtained results allowed qualitative or at best semiquantitative statements. A preincubation step of RBD and serum, as performed for the assay using RBD-liposomes, can potentially enhance sensitivity but is by itself insufficient. The strength of these colorimetric assays is the potential for analysis via smartphone. To provide meaningful data with qualitative statements, the assays would need to be tuned to a CoP. Tong et al. managed to develop a quantitative colorimetric LFNT based on the use of RBD-modified polydopamine nanoparticles.^115^ These needed to be coated by three polyelectrolytes, a SiO_2_, and a poly(ethylene glycol) (PEG) layer to prevent nonspecific binding, making particle formation rather complex. Their deep-learning algorithm enabled reliable detection with different smartphones, and they could show good correlation to a commercial ELISA (*r*_S_ = 0.951) and better performance than a AuNP-based LFNT.

**Table 3:** List of Colorimetric and Fluorescence LFNTs

capture protein (immobilized)	target protein	signaling conjugate	comments and correlation to other neutralization tests	incubation steps and time	ref
AuNP-Based Colorimetric LFNTs					
ACE2	RBD-AuNPs	RBD-AuNPs	multiplexing (delta and omicron) ELISA, *R*^2^ = 0.8777, *n* = 21	15 min	(107)
ACE2	RBD-AuNPs	RBD-AuNPs	pVNT, *r* = 0.918, *n* = 165	not mentioned	(119)
ACE2	RBD-biotin	antibiotin-AuNPs	additional RBD test line for anti-RBD-Ig microNT, *R*^2^= 0.72, *n* = 135	20 min	(109)
ACE2 (5 spots)	RBD-biotin	streptavidin-AuNPs	laser-cut narrow membrane no correlation	2 min + 15 min	(108)
ACE2	Spike-biotin	streptavidin-AuNPs	WHO standard and 10 sera tested, only compared to commercial binding antibody test	15 min	(110)
					
Other Colorimetric LFNTs					
RBD-CBD	biotinylated monoFc-ACE2	streptavidin-HRP (+ TMB)	paper-based cellulose pull-down assay, smartphone read-out pVNT, *r* = 0.7, *n* = 48	5 min + 3 min	(111)
ACE2	RBD-nanoshells	RBD-nanoshells	microNT, *n* = 38, grouped by IC50 values	10 min	(112)
ACE2	RBD	anti-RBD-cellulose nanobeads	also works as antigen test when used without free RBD 10 seropositive and 5 seronegative samples tested, not correlated	20 min	(113)
ACE2	RBD-red latex microbeads	RBD-red latex microbeads	not correlated	9 min	(114)
ACE2	RBD-liposomes encapsulating SRB	RBD-liposomes encapsulating SRB	dual use for colorimetric LFA and fluorescence microplate HTS assay pVNT, *r*_S_ = 0.614, *n* = 20	15 min + 25 min	(90)
ACE2	RBD-PVP@SiO_2_ @PEG@Ab NPs	RBD-PVP@SiO_2_ @PEG@Ab NPs	ELISA, *r*_S_ = 0.951, *n* = 30	20 min	(115)
					
Fluorescence LFNTs					
ACE2	RBD-EuNPs	RBD-EuNPs	cVNT, 88.76% coincidence rate, 216 seronegative and 140 seropositive samples tested	20 min	(116)
RBD-CBD	Alexa Fluor 594-labeled monoFc-ACE2	Alexa Fluor 594-labeled monoFc-ACE2	paper-based; venous and finger prick blood; adapted for variants pVNT, *r* = 0.91, *n* = 20; cPass, *r* = 0.839, *n* = 44	3 min + 8 min	(117)
RBD	ACE2-ultrabright AIE490NP	ACE2-ultrabright AIE490NP	correlation of fluorescence-quenching LFNT in follow-up publication pVNT, *R*^2^ = 0.9796, *n* = 103	20 min	(118)
					(120)
ACE2	RBD-biotin	QD-streptavidin	ELISA, *R*^2^ = 0.85, *n* = 40 pVNT, *R*^2^ = 0.53, *n* = 40	10 min	(121)

To overcome the issue of sensitivity, some groups turned to a fluorescence read-out, as seen more frequently in the LFA field recently. The use of EuNPs conjugated to RBD enabled a quantitative read-out within 20 min with a fluorescence ICS card reader.^116^ The Alexa Fluor 594-labeled monoFc-ACE2 based assay of Lim et al. gave semiquantitative results within 10 min, is adaptable to multiple variants, and works with both venous and finger prick blood.^117^ Most fluorophores show quenching when in an aggregated state, which is promoted when captured on the test line, resulting in lower sensitivity. Aggregation-induced emission (AIE) luminogens, on the other hand, can benefit from the close proximity of the molecules. Bian et al. encapsulated AIE490 in polystyrene nanoparticles and observed a 10-fold increase of fluorescence compared to free AIE490; the AIE490-NPs were even brighter than quantum dots.^118^ Modified with ACE2, the particles facilitated semiquantitative detection of nAbs. The limit of detection (LOD) was 4 times lower compared to a colorimetric LFA using AuNPs. Slight modification of the assay to obtain a fluorescence-quenching read-out could further improve the LOD 9-fold and will be discussed in more detail in the chapter dealing with signal-on strategies.

### Other Detection Methods

Other read-outs included NIR,^122^ magnetoresistive,^123^ and thermal and Raman detection,^41^ as well as particle counting.^124^ NIR is interesting as it benefits from low background signals in biological matrices. Song et al. even developed a hand-held NIR detection device that was 8 times more sensitive than its commercial counterpart.^122^ They used Nd^3+^- and Yb^3+^-codoped down-conversion nanoparticles, coated with poly(acrylic acid) to enable EDC/sulfo-NHS coupling of RBD and an ACE2 test line to facilitate read-out within 15 min. Their assay was in qualitative agreement with a commercial ELISA for alpha and omicron variants as investigated using 50 sera, but no correlation to an established neutralization test was provided. The giant magnetoresistive (GMR) neutralization test developed by Ng et al. provides an alternative read-out strategy but currently relies on subsequent 1 h incubation steps of serum with RBD-biotin and on the ACE2-chip including washing, necessitating simplification before the assay is ready for the POC.^123^ Generally, GMR is an attractive alternative for conventional POC detection as biological samples are nonmagnetic, making the assays virtually background-free, and hand-held and automated POC readers have been established.^125,126^ Zhao at el. used AgNPs with Au shell, a PEG-COOH layer allowing for conjugation of the S protein, and an LFA test strip with an ACE2 test line. They compared visual, photothermal, and SERS read-outs.^41^ The thermal camera captured images during irradiation with an 808 nm laser (2 W/cm^2^) and provided 10 times more sensitive results compared to the visual detection. A portable Raman spectrometer with 785 nm laser produced quantitative results identical with those of photothermal detection. This combination of technologies should be investigated more for its applicability for the POC because it could provide a means for qualitative visual read-out by laypersons with the option for quantitative read-out, e.g., by mobile health clinics in areas with limited resources.

### Signal-On Strategies

All of the tests described above are “signal-off” strategies because they are based on signal generation in the absence of neutralizing antibodies. Low titers are thus difficult to quantify, as the resulting decrease of bound conjugates might be too small to be measured. Consequently, the tests have poor resolution in the lower and good resolution in the upper nAb titer range. Thus, “signal-on” strategies have been investigated addressing this issue. Besides improved sensitivity, this can make the tests more user-friendly, with increasing signal intensities correlating to increasing and not decreasing antibody titers and thus immunity.

**Figure 7:**
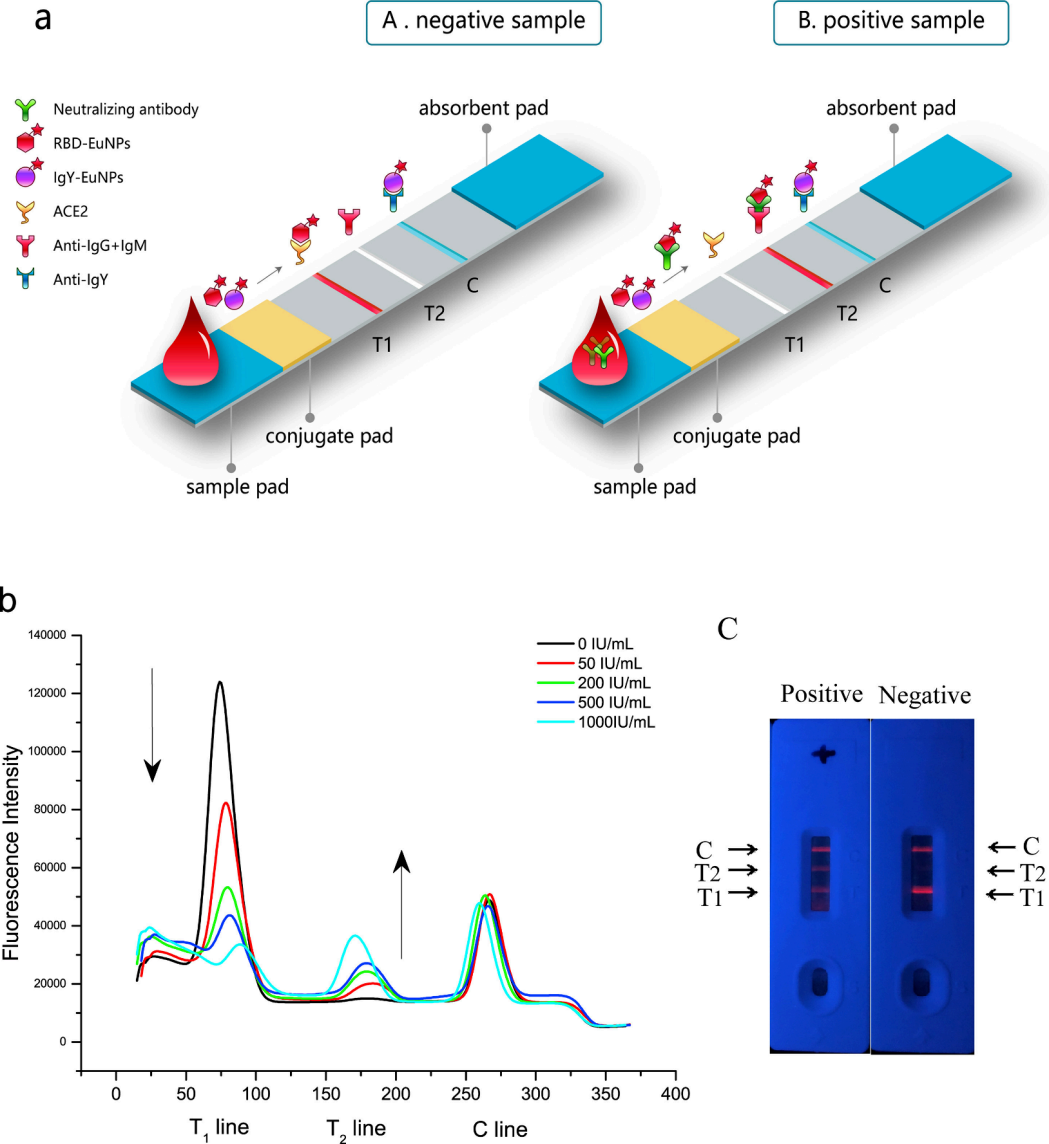
(a) Schematic of the EuNP-based signal-on LFNT. (b) Inverse relationship of the fluorescence signals toward different concentrations of neutralizing antibody. (c) Positive and negative samples tested using the LFNT cassette. Reprinted with permission from ref (128). Copyright 2022 Elsevier.

The straightforward approach is to try to capture the neutralized RBD or S protein conjugates. This was done by either an antihuman IgG,^127^ antihuman IgG + IgM + IgA,^128^ or protein A^129^ in addition to an ACE2 test line (Figure 7a). However, these capture all antibodies of the targeted isotype and not only anti-RBD/Spike antibodies. This can be seen by the smaller signal increase for neutralized compared to the signal decrease for a non-neutralized conjugate (Figure 7b). Rather than relying solely on the signal of the neutralized test line, the tests use the ratio of neutralized to non-neutralized test line signals for quantification of nAbs. With total IgG and IgM levels around 10 and 1 g/L, respectively,^130^ it becomes clear that these test lines need to be highly concentrated to capture sufficient antibodies, while sample volumes have to be kept low to avoid overloading them. Connelly et al. went for a rather high 1:800 dilution of plasma or whole blood, starting out with 1 μL.^129^ Only 150 μL of this dilution was then added to an RBD-conjugated 5 mm circular-punched colloidal gold pad. Finally, 75 μL of this solution was used for the LFA, corresponding to only 0.14 nL of the original sample. At such low concentrations, it would be expected that only the most potent seropositive samples, if any, could be detected. Nonetheless, they showed the successful capture of neutralized conjugates.

Duan et al. used 10 μL of serum or 20 μL of whole blood, as did most of the previously discussed LFNTs, for their EuNP-based test and obtained an area under the ROC curve of 0.955 for 266 samples when compared with the cPass sVNT.^128^ Their results were also in good agreement with the WHO reference panel. Using an antihuman Ig test line, his-RBD, and anti-his-antibody-modified quantum dots, Li et al. also required 10 μL of patient sample.^127^ The assay showed excellent correlation to ELISA and good correlation to pVNT. Two other groups presented “signal-on” strategies with RBD test lines. Zhang et al. developed a double-antigen sandwich LFA using RBD-conjugated latex beads (LB) or fluorescence beads (FB).^131^ They screened nine types of RBD as antigen pairs for the LB and four types for the FB, immobilizing one as test line and conjugating the other to the beads, and chose the combinations that resulted in the lowest signal for a seronegative and the highest signal for a seropositive sample. The optimized systems showed excellent sensitivity and specificity compared to a live-virus neutralization test, as investigated with serum panels consisting of 389 (FB) and 554 (LB) samples. It unfortunately remains unclear what the difference between the nine investigated RBDs is and whether adaptation to other variants is feasible or would require expression and screening of multiple RBD types once again. Bian et al. used the previously discussed AIE490NPs conjugated to RBD or BSA as test lines.^120^ Upon binding of ACE2-AuNPs to the RBD-AIE490NPs, the fluorescence of the latter is quenched. In the absence of neutralizing antibodies, the assay shows the maximum colorimetric signal for their AuNPs and the minimum fluorescence signal for the RBD-AIE490NP test line. The BSA-AIE490NPs serve as reference, providing the maximum obtainable fluorescence signal. The fluorescence-quenching LFNT showed excellent correlation to a pVNT (*R*^2^ = 0.9796, *n* = 50) and intra- and interassay precisions below 15%.

## Conclusions and Perspectives

Serological testing continues to be an important tool for the understanding of the immune response and fight against diseases. While binding antibody tests and cell-based neutralization tests have been established for different viruses, the recent SARS-CoV-2 pandemic has brought the development of surrogate virus neutralization tests into focus. These promise to fill the gap for rapid, cost-efficient, and large-scale assessment of the immune status of large parts of the population. Much development has occurred within the last 5 years, focusing on high-throughput screening and POC testing, and many products have reached the market. However, federal agencies such as the U.S. FDA, CDC, NIH, and ECDC have not endorsed their use to assess the need for booster vaccinations due to lack of validation and standardization. Furthermore, the rapid genetic drift of the virus highlights the need for test adaptability while complicating studies to define specific neutralizing antibody titers that work as CoP, which, in turn, requires screening of large serum panels. The sVNTs discussed in this review include many valuable variations of commercialized assay formats as well as novel ones that can improve sensitivity and throughput, reduce assay time and cost, and enable multiplexing. Important advancements for HTS assays include the automated use of microarrays and Luminex technology for multiplexing and assay simplification achieved by homogeneous platforms enabled, e.g., by the use of split-NanoLuc strategies. For POC detection, several multimodal systems have been developed that could make sensitive serological testing more widely available with the option for qualitative visual or quantitative read-out using portable detectors. It is important to note that at this point there is still no single assay that fulfills all criteria claiming superiority to all the others; rather, each assay has their own strengths and weaknesses. Many choices have to be made prior to and during assay development, including decisions on especially assay and sample type, the desired sensitivity, and the option of adaptation to other variants or viruses.

Due to the pandemic, most advancements in sVNT technology were made based on SARS-CoV-2 as the model analyte; however, because the tests need to be adapted to virus variants, they can just as easily be adapted to other viruses altogether. The WHO Pathogens prioritization framework from July 2024 lists bacteria and 29 families of viruses, of which 14 are assigned with a low risk, 2 with a low-to-medium risk, 2 with a medium risk, and 12 with a high risk for causing Public Health Emergencies of International Concern (PHEICs).^132^ The last category includes Poxviridae as the only family of DNA viruses and 10 families of RNA viruses, which include many well-known viruses such as ebola, dengue, influenza, monkeypox, nipah, zika, and chikungunya. The list is subject to change because climate change, deforestation, urbanization, international travel, and other major global changes can have a direct or indirect influence on the spread of viruses.^133^ Serological testing with PRNTs and pVNTs has been conducted for many of these viruses and, in some cases, identified neutralizing antibodies as CoP, as is the case for SARS-CoV-2, nipah, and ebola according to Escudero-Pérez et al.^134^ Neutralizing antibodies detected using a PRNT or a flow-cytometry-based neutralization test were also found to work as potential CoP for chikungunya^135^ and dengue viruses,^136^ respectively. Because such a correlation has been identified, further simplification of the assay could be useful if large-scale screening outside of BSL facilities is desired, e.g., to assist with the licensing of vaccines.^137^ This purpose could be served by sVNTs, given that they are properly validated with the use of standard reference serum panels, and the results are found to be in agreement to those of the PRNT.

It is important to consider the potential targets of neutralizing antibodies for each virus to evaluate the possibility for sVNT development. The major target is often the receptor binding protein of the virus,^138−140^ which is responsible for the interaction between the virus and host cell, but others such as the envelope protein might also be of importance.^141,142^ For viruses in the Paramyxoviridae and Orthomyxoviridae families, which includes influenza, neutralizing antibodies target the hemagglutinin glycoproteins, which bind to sialic acid residues on the host cell.^143^ Thus, conjugation strategies for both small molecules and proteins would be required. If the targeted protein is subject to frequent mutation, this has to be accounted for regarding the choice of conjugation strategy, which is often directly intertwined with protein expression. Multiplexing for several variants could be of interest in this case and of higher importance than the need for POC application. In general, there are many variables that need to be taken into account when developing sVNTs, which are best addressed by the collaboration of experts from different fields including virology, bioanalytical chemistry, and clinicians. In the end, the technology advancement in HTS and POC for neutralizing antibody detection will, furthermore, affect our ability to detect many other biomarkers that are based on protein–protein interactions. This can be exploited in drug development, in biomarker-based companion diagnostic and precision medicine, and also in the fields relating to prevention such as the secondary health market, lifestyle, nutrition, and food safety applications.
